# Childhood maltreatment and personality disorders in adolescents and adults with psychotic or non-psychotic disorders

**DOI:** 10.3389/fpsyt.2024.1336118

**Published:** 2024-03-21

**Authors:** WenZheng Wang, Yin Cui, Qiang Hu, YanYan Wei, LiHua Xu, XiaoChen Tang, YeGang Hu, HaiChun Liu, ZiXuan Wang, Tao Chen, Ran Wang, CuiXia An, JiJun Wang, TianHong Zhang

**Affiliations:** ^1^ Shanghai Mental Health Center, Shanghai Jiaotong University School of Medicine, Shanghai Engineering Research Center of Intelligent Psychological Evaluation and Intervention, Shanghai Key Laboratory of Psychotic Disorders, Shanghai, China; ^2^ Department of Psychiatry, ZhenJiang Mental Health Center, Zhenjiang, China; ^3^ Department of Automation, Shanghai Jiao Tong University, Shanghai, China; ^4^ Shanghai Xinlianxin Psychological Counseling Center, Shanghai, China; ^5^ Big Data Research Lab, University of Waterloo, Waterloo, ON, Canada; ^6^ Labor and Worklife Program, Harvard University, Cambridge, MA, United States; ^7^ Department of Psychiatry, The First Hospital of Hebei Medical University, Shijiazhuang, Hebei, China; ^8^ Hebei Technical Innovation Center, Mental Health Assessment and Intervention, Shijiazhuang, Hebei, China; ^9^ Hebei Clinical Research Center of Mental Disorders, Institute of Mental Health, Shijiazhuang, Hebei, China; ^10^ Institute of Psychology and Behavioral Science, Shanghai Jiao Tong University, Shanghai, China; ^11^ Center for Excellence in Brain Science and Intelligence Technology (CEBSIT), Chinese Academy of Science, Shanghai, China

**Keywords:** personality traits, trauma, childhood abuse, childhood neglect, age

## Abstract

**Introduction:**

While the attention to personality disorders (PD) and childhood maltreatment (CM) has grown in recent years, there remains limited understanding of the prevalence and distinctions of PD and CM in clinical populations of Chinese adolescents in comparison to adults.

**Methods:**

A total of 1,417 participants were consecutively sampled from patients diagnosed with either psychotic or non-psychotic disorders in the psychiatric and psycho-counseling clinics at Shanghai Mental Health Center. The participants were categorized into two groups based on their age: adolescents (aged 15-21 years) and adults (aged 22-35 years). PDs were evaluated using a self-reported personality diagnostic questionnaire and a structured clinical interview, while CMs were assessed using the Chinese version of the Child Trauma Questionnaire Short Form.

**Results:**

When comparing self-reported PD traits and CM between adolescents and adults, differences emerge. Adolescents, particularly in the psychotic disorder group, exhibit more pronounced schizotypal PD traits (*p*=0.029), and this pattern extends to non-psychotic disorders (*p*<0.001). Adolescents in the non-psychotic disorder group also report higher levels of emotional abuse (*p*=0.014), with a notable trend in physical abuse experiences compared to adults (*p*=0.057). Furthermore, the most prevalent PDs in the clinical sample are avoidant, borderline, and obsessive-compulsive PDs. Among patients with psychotic disorders, adolescents exhibit higher rates of schizoid, schizotypal, and obsessive-compulsive PDs compared to adults. Logistic regression analyses highlight distinct predictors for psychotic and non-psychotic disorders in adolescents and adults.

**Discussion:**

The findings emphasize distinct differences in PDs and CMs between adolescent and adult groups, shedding light on their potential roles in psychotic and non-psychotic disorders.

## Introduction

Personality disorders (PDs) are relatively common among patients with psychiatric disorders (1–3), and specific types of PDs may serve as susceptibility factors for certain mental disorders (4, 5). High comorbidity rates exist between PDs and mental disorders (6, 7). For instance, our previous research has indicated that features of schizotypal PD can play a role in identifying individuals at clinical high risk for psychosis (8). Furthermore, the distribution of both PD traits and psychiatric symptoms varies significantly across different age groups (9, 10). Since the PD construct continues to be controversial when applied to adolescents (11), there has been limited research on PD features among adolescent patients. This is especially true in the context of the assessment and diagnosis of PDs within clinical populations in China (12). As a result, there is currently limited data available to compare the features of PDs in adolescent patients with those in adults.

Childhood maltreatment (CM) is recognized as a dual-risk factor, significantly contributing to both PDs and mental disorders (13–15). The intricate interplay between CMs and these conditions emphasizes the complex and interconnected nature of these factors (16–18). However, it is crucial to consider the impact of age on the experience and self-reporting of CMs, with particular emphasis on the potential differences between adolescents and adults. Adolescents, due to their evolving cognitive and emotional capacities, as well as changing social dynamics, may possess distinct experiences and disclosure patterns for CMs in comparison to adults (19). These age-related disparities could be attributed to the unique developmental stages and socio-cultural influences that shape the way adolescents perceive and report their CM experiences (20, 21). Additionally, the impact of CM on adolescents might be more direct, given their closer proximity to the experiences, potentially exacerbating its effects on psychological well-being. Thus, exploring the manifestations of CM and PDs in adolescents compared to adults can provide valuable insights into the developmental trajectories of mental health disorders.

Expanding upon the previously mentioned context, this study aims to explore and contrast self-reported PD traits and CM experiences among male and female individuals diagnosed with SZ. Furthermore, we seek to evaluate the prevalence of PD diagnoses through structured interviews within different gender groups. Our study hypothesis suggests the existence of sex differences in PD pathology and CM within the population of individuals diagnosed with SZ.

## Methods

### Participants

The survey was carried out between 2018 and 2019 at the Shanghai Mental Health Center (SMHC), one of China’s largest healthcare facilities. Participants were selected from the pool of outpatients attending psycho-counseling clinics and psychiatric clinics at SMHC. The study involved a consecutive clinical sample of adult patients aimed at examining the prevalence of both PDs and CMs. A total of 1,417 randomly selected outpatients were enrolled from January 2018 to December 2019. Inclusion criteria consisted of patients within the age range of 15 to 30 years who could comprehend the study questionnaire, were willing to disclose information about PDs and CMs, and were under stable treatment conditions. Exclusion criteria encompassed severe or unstable physical conditions, such as advanced stages of cancer, severe organ failure, debilitating chronic diseases (e.g., severe heart failure), uncontrolled hypertension, unstable angina, or acute infections, current pregnancy, and any other factors identified by the investigators as rendering the patient ineligible. Following a meticulous process of double data entry and verification, 1,417 patients were included in the analysis.

### Measurements

#### General questionnaire

The general questionnaire gathered data in the following categories: (a) demographic information; (b) family and social background; and (c) physical and mental health status,

#### Clinical diagnoses

The primary clinical diagnoses were determined following the criteria outlined in the Chinese Classification of Mental Disorders Third Edition (CCMD-3), and these diagnoses were conducted by attending psychiatrists in outpatient settings, who typically have at least five years of clinical experience specializing in psychiatry. The diagnostic categories within CCMD-3 were influenced by the DSM-IV and ICD-10, with the majority of diagnostic criteria for disorders, including psychotic disorders (such as schizophrenia, paranoid mental disorders, and schizoaffective psychosis), as well as non-psychotic disorders like mood disorders (including mania, bipolar disorder, depression, and dysthymia) and anxiety disorders (including phobia, panic disorder, generalized anxiety disorder, and OCD), being either identical or closely aligned with international classification systems.

#### PDs assessment

A succinctly structured self-report questionnaire, the personality diagnostic questionnaire 4^th^ edition plus (PDQ-4+) (22) as detailed in our previous works (8, 10, 23), is employed. This questionnaire comprises 107 true-false questions and is designed to assess 10 Axis II DSM-IV PDs, including Paranoid (PAR), Schizoid (SCH), Schizotypal (SCHT), Histrionic (HIS), Narcissistic (NAR), Borderline (BOR), Antisocial (ANT), Avoidant (AVO), Dependent (DEP), Obsessive-compulsive (OBC). The objective of the PDQ-4+ is to differentiate individuals with characteristics associated with PD from those without. The PDQ-4+ exhibits high sensitivity (0.89) and acceptable specificity (0.65). It has been utilized for screening DSM-IV PD in Chinese psychiatric patients. The high test-retest reliability score (0.92) within the Chinese population underscores the reliability of the questionnaire’s results (12).

The Structured Clinical Interview for DSM-IV Axis II (SCID-II), which is a semi-structured clinical interview for diagnosing PDs, employed DSM-IV criteria for the classification of personality disorders. Our team translated and implemented the Chinese version of SCID-II. The results obtained using SCID-II exhibit a high level of consistency (0.90) with clinical diagnoses, and the test-retest reliability is also satisfactory (0.70) (24). The SCID-II assessments were conducted by trained research personnel, who have a minimum of two years of professional experience and received specific training in administering the SCID-II.

#### CMs assessment

CMs were assessed using the Chinese version of the Child Trauma Questionnaire Short Form (CTQ-SF) (25–27). The CTQ-SF consists of 28 self-report items that are categorized into five childhood maltreatment subscales: emotional abuse, physical abuse, sexual abuse, emotional neglect, and physical neglect. Participants rated the frequency of each event on a 5-point scale, ranging from 1 (never) to 5 (always), with higher scores indicating a greater degree of CMs. The Chinese version of the CTQ-SF has been established as a reliable and valid tool for evaluating CMs within Chinese clinical samples (26, 28, 29).

### Statistical analyses

SPSS for Windows (version 20.0) was used for data analysis. Statistical significance was set at p<0.05. Quantitative variables are expressed as mean ± standard deviation (SD), and qualitative variables as frequencies (%). Participants were divided into adolescent and adult groups according to age: 15-21 years and 22-35 years (30). A logistic regression model was fitted to identify factors associated with clinical diagnosis. Variables in the logistic models were selected based on the sex, 10 subtypes of PDs, 5 subtypes of CMs. We reported the β according to 95% confidence intervals (CI) and P-values of Wald tests for the logistic models.

## Results

The sociodemographic and basic clinical information of the 1417 participants is presented in **Table 1**. The participants’ ages ranged from 15 to 30 years, with a mean age of 22.0 ± 4.278 years. There were 671 (47.4%) men and 746 (52.6%) women, demonstrating a generally equal distribution. The percentage of individuals with a college education or higher was higher among adults than among adolescents. Additionally, the average duration of illness was longer in adults than in adolescents.

**Table 1:** Socio-demographic characteristics for patients with schizophrenia.

	Adolescents		Adults		*χ^2^*/Z	*p*
	N/Means	%/SD.	N/Means	%/SD.		
Cases	674		743		-	-
Gender						
Male	329	48.8%	342	46.0%	1.098	0.295
Female	345	51.2%	401	54.0%		
Age (year)	18.2	1.972	25.4	2.570	-	-
Range for Age (year)	15-21		22-35		-	-
Diagnosis						
Psychotic disorder	198	29.4%	208	28.0%	0.330	0.565
Non-Psychotic disorder	476	70.6%	535	72.0%		
Education						
Middle or high school	377	55.9%	338	45.5%	**15.419**	**<0.001**
College or higher	297	44.1%	405	54.5%		
Self-reported Pre-illness characteristic						
Introversion	296	43.9%	298	40.1%	2.233	0.327
Middle type	271	40.2%	314	42.3%		
Extroversion	107	15.9%	131	17.6%		
Family history of mental disorder						
With family history	60	8.9%	90	12.1%	3.850	0.050
Without family history	614	91.1%	653	87.9%		
Visits and Course						
First Visit	399	59.3%	431	58.1%	7.587	0.055
2-5 visits	162	24.0%	157	21.1%		
6-10 visits	46	6.8%	47	6.3%		
>10 visits	67	9.9%	108	14.5%		
Course of illness (months)	27.3	29.421	46.4	48.989	**-4.757**	**<0.001**
Range for the course of illness (months)	1-84		1-240		**-**	**-**

Z values for nonparametric tests using the Mann-Whitney U test. Significant values are indicated in bold.

**Tables 2** and **3** compare the differences in self-reported PD traits and CMs between the adolescent and adult groups. The results show that in the psychotic disorder group, SCHT PD traits are more pronounced in the adolescent group. In the non-psychotic disorder group, SCHT and ANT traits remain more pronounced in the adolescent group. Adolescents in the non-psychotic disorder group also reported a higher prevalence of EA experiences, and reported PA experiences tend to be significant (p=0.057) than adults.

**Table 2:** Self-reported PD traits in patients with psychotic and non-psychotic disorders, stratified by adolescents and adults.

PDs	Adolescents		Adult		Z	p
	Mean	SD.	Mean	SD.		
Psychotic disorder						
PAR	3.25	1.605	3.30	1.705	-.168	.867
SCH	2.69	1.375	2.65	1.446	-.635	.525
SCHT	4.73	1.835	4.29	2.011	**-2.184**	**.029**
ANT	2.28	1.602	2.03	1.672	-1.560	.119
BOR	4.76	2.173	4.72	2.385	-.380	.704
HIS	3.85	1.792	3.97	1.876	-.632	.528
NAR	3.70	2.045	3.71	1.994	-.139	.889
AVO	4.40	1.634	4.11	1.744	-1.699	.089
DEP	3.76	1.847	3.92	1.893	-.796	.426
OBC	4.30	1.855	4.01	1.873	-1.489	.136
Non-Psychotic disorder						
PAR	3.40	1.778	3.46	1.780	-.533	.594
SCH	2.66	1.624	2.60	1.579	-.534	.593
SCHT	4.49	1.948	4.09	1.908	**-3.630**	**.000**
ANT	2.24	1.704	1.99	1.705	**-2.544**	**.011**
BOR	5.23	2.157	5.22	2.156	-.137	.891
HIS	4.11	1.740	4.11	1.722	-.143	.886
NAR	3.95	1.942	3.88	1.918	-.373	.709
AVO	4.37	1.800	4.44	1.717	-.573	.567
DEP	3.87	1.973	3.94	1.959	-.542	.588
OBC	4.30	1.668	4.40	1.708	-1.102	.271

Z values for nonparametric tests using the Mann-Whitney U test. PAR, Paranoid PD; SCH, Schizoid PD; SCHT, Schizotypal PD; HIS, Histrionic PD; NAR, Narcissistic PD; BOR, Borderline PD; ANT, Antisocial PD; AVO, Avoidant PD; DEP, Dependent PD; OBC, Obsessive-compulsive PD. Significant values are indicated in bold.

**Table 3:** Self-reported childhood maltreatment (CM) in patients with psychotic and non-psychotic disorders, stratified by adolescents and adults.

CMs	Adolescents		Adult		*Z*	*p*
	Mean	SD.	Mean	SD.		
Psychotic disorder						
EA	8.08	3.168	7.74	3.169	-1.388	.165
PA	6.57	2.408	6.41	2.625	-1.218	.223
SA	6.14	2.175	6.39	2.403	-.962	.336
EN	17.93	4.767	17.99	4.320	-.569	.569
PN	9.24	2.884	9.20	3.157	-.228	.820
Non-Psychotic disorder						
EA	8.40	3.675	7.78	3.243	**-2.461**	**.014**
PA	6.61	2.736	6.27	2.241	-1.907	.057
SA	5.88	1.912	5.74	1.823	-1.499	.134
EN	18.63	5.127	18.46	4.979	-.475	.634
PN	8.86	3.119	8.76	3.223	-.756	.450

Z values for nonparametric tests using the Mann-Whitney U test. EA, Emotional abuse; PA, Physical abuse; SA, Sexual abuse; EM, Emotional neglect; PN, Physical neglect. Significant values are indicated in bold.

In addition to examining differences in PD traits between probands with (n=150) and without (n=1267) a family history of mental illness, we also investigated CM experiences. Our analysis revealed that probands with a family history reported significantly higher levels of PA (*t*=2.313, *p*<0.05), SA (*t*=3.292, *p*<0.05), and EN (*t*=2.392, *p*<0.05) compared to those without a family history.

**Figure 1:**
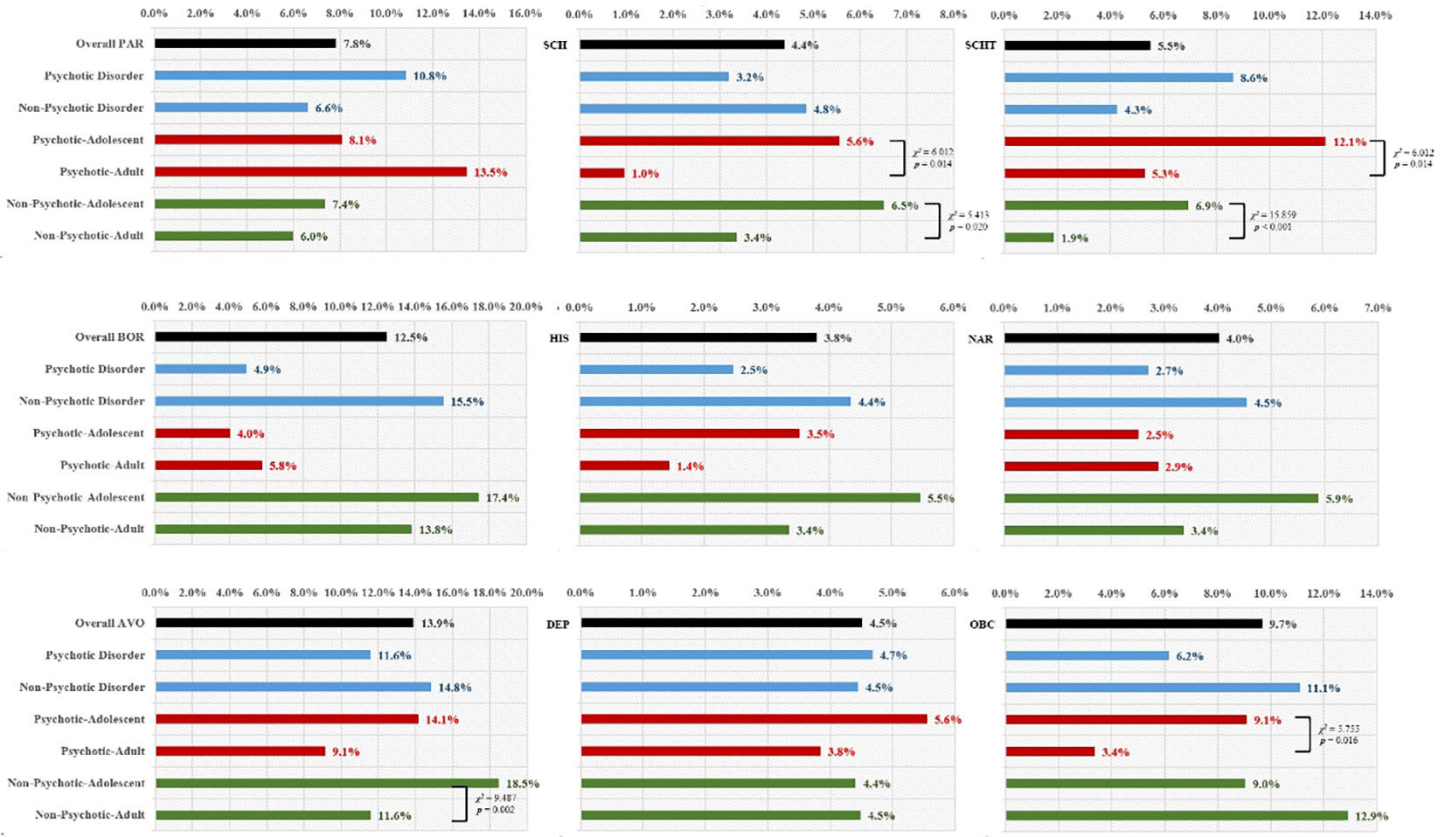
Frequency of Personality Disorders (PDs) in Patients with Psychotic and Non-psychotic disorders, Stratified by Adolescents and Adults. PAR, Paranoid PD; SCH, Schizoid PD; SCHT, Schizotypal PD; HIS, Histrionic PD; NAR, Narcissistic PD; BOR, Borderline PD; ANT, Antisocial PD; AVO, Avoidant PD; DEP, Dependent PD; OBC, Obsessive-compulsive PD.

As shown in **Figure 1**, in the clinical sample of this study, AVO, BOR, and OBC were the three most common types of PDs. Among patients with psychotic disorders, SCH, SCHT, and OBC PDs were more common in adolescents than in adults. In the Non-Psychotic disorder group, SCH, SCHT, and AVO PDs were more common in adolescents than in adults.

Logistic regression analyses were conducted with the diagnosis as the dependent variable, and gender, different types of self-reported PDs, and CMs as independent variables (**Table 4**). In adolescents, being male, having higher scores on the SCHT and ANT PDs, and experiencing EN were significant predictors of psychotic disorders, while being female, having a higher self-reported score on the BOR PD, and experiencing PN were significant predictors of non-psychotic disorders. In adults, having a higher self-reported score on SCHT and SA were significant predictors of psychotic disorders, while having a higher score on the BOR PD was a significant predictor of non-psychotic disorders.

**Table 4:** Logistic regression analysis of childhood maltreatment (CM) and personality disorder (PD) factors predicting clinical diagnoses, stratified by adolescents and adults.

Variables	Analysis						
	Beta	S.E.	*β*	95%CI for *β*		*Wald*	*P*
Adolescents							
Sex (male)	.413	.183	1.511	1.055	2.165	5.076	**.024**
PAR	.038	.067	1.038	.911	1.184	.317	.573
SCH	.018	.061	1.018	.903	1.148	.085	.771
SCHT	-.200	.062	.819	.724	.925	10.286	**.001**
ANT	-.130	.064	.878	.775	.995	4.160	**.041**
BOR	.195	.057	1.215	1.087	1.358	11.817	**.001**
HIS	.034	.062	1.035	.916	1.169	.304	.581
NAR	.068	.062	1.070	.947	1.209	1.188	.276
AVO	-.072	.062	.931	.825	1.051	1.341	.247
DEP	.018	.054	1.018	.916	1.131	.110	.740
OBC	-.020	.059	.980	.873	1.100	.119	.730
EA	.054	.035	1.055	.985	1.131	2.318	.128
PA	-.032	.041	.969	.894	1.050	.606	.436
SA	-.072	.045	.930	.852	1.016	2.570	.109
EN	.053	.022	1.055	1.011	1.101	5.978	**.014**
PN	-.077	.034	.926	.866	.990	5.105	**.024**
Adults							
Sex (male)	.167	.178	1.182	.834	1.675	.881	.348
PAR	.030	.062	1.030	.912	1.164	.234	.629
SCH	-.024	.060	.976	.868	1.098	.161	.688
SCHT	-.175	.059	.839	.747	.942	8.776	**.003**
ANT	-.074	.067	.928	.814	1.058	1.236	.266
BOR	.164	.056	1.179	1.056	1.315	8.638	**.003**
HIS	.028	.063	1.028	.910	1.163	.201	.654
NAR	-.014	.061	.986	.875	1.111	.053	.818
AVO	.118	.062	1.125	.995	1.272	3.561	.059
DEP	-.074	.056	.928	.831	1.037	1.735	.188
OBC	.112	.058	1.118	.998	1.253	3.724	.054
EA	.049	.037	1.050	.977	1.130	1.757	.185
PA	-.021	.045	.979	.896	1.070	.218	.641
SA	-.155	.046	.856	.783	.937	11.466	**.001**
EN	.029	.022	1.029	.986	1.074	1.721	.190
PN	-.051	.031	.951	.894	1.010	2.644	.104

Beta is the regression coefficient. S.E. is the standard error. 95% CI is the estimated 95% confidence interval for the corresponding parameter. β is the standardized regression coefficient. PAR, Paranoid PD; SCH, Schizoid PD; SCHT, Schizotypal PD; HIS, Histrionic PD; NAR, Narcissistic PD; BOR, Borderline PD; ANT, Antisocial PD; AVO, Avoidant PD; DEP, Dependent PD; OBC, Obsessive-compulsive PD; EA, Emotional abuse; PA, Physical abuse; SA, Sexual abuse; EM, Emotional neglect; PN, Physical neglect. Significant values are indicated in bold.

## Discussion

Our study revealed noteworthy differences between adolescent and adult groups in terms of self-reported PD traits and childhood maltreatment experiences. Among adolescents with psychotic disorders, SCHT PD traits were more prominent, while in the non-psychotic disorder group, SCHT and ANT PD traits remained pronounced among adolescents. Additionally, adolescents in the non-psychotic disorder group reported higher levels of EA experiences, with significant trends in reported PA experiences. The most prevalent PDs in our clinical sample were AVO, BOR, and OBC PDs, with variations between age groups. Logistic regression analyses further emphasized the predictive factors for psychotic and non-psychotic disorders, with distinct patterns in adolescents and adults. This study underscores the significance of age-related differences in the presentation of PD traits, childhood maltreatment, and their implications for psychiatric diagnoses. To the best of our knowledge, this represents the first extensive comparative survey in a clinical Chinese population, examining the frequency of PD and CM in both adolescents and adults.

The observed disparities in self-reported PD traits between adolescents and adults are noteworthy. In the case of adolescents with psychotic disorders, SCHT PD trait was found to be more prominent. Similarly, among adolescents in the non-psychotic disorder group, both SCHT and ANT PD traits exhibited greater prominence compared to their adult counterparts. One potential explanation for these differences may lie in the identity formation that occur during adolescence. Adolescence is a critical period for identity formation (31), where individuals experiment with various roles, beliefs, and behaviors. SCHT PD traits, which include eccentric beliefs and unusual perceptual experiences (8), might resonate with the exploratory nature of this developmental phase. Adolescents may be more open to expressing such traits as part of their identity exploration. Some mental health conditions, such as schizophrenia, have an earlier onset during adolescence (32, 33). The overlap of symptoms between SCHT PD traits and schizophrenia may lead adolescents to self-report these traits when experiencing early signs of psychotic disorders, thus making them more prominent. Adolescents are highly influenced by their peer groups, which can sometimes involve risky behaviors and impulsivity. These characteristics are core features of ANT PD traits (34). Adolescents might be more inclined to report these traits, especially when trying to fit in with certain social groups or navigate the peer pressures of their age group.

The differences observed in self-reported CM experiences between adolescents and adults, particularly the higher levels of EA and the significant trends in PA in the adolescent group, can be attributed to various factors. Adolescence is a crucial period for emotional and cognitive development. During this stage, individuals may become more aware of past traumatic experiences, including EA and PA (26, 35). The increased ability to reflect on and report these experiences may result in a higher likelihood of self-reporting. Adolescents often experience heightened emotional intensity, which can lead to a deeper understanding of their emotional experiences, including those related to EA. This increased emotional awareness may prompt adolescents to report these experiences. Besides, adolescents may become more willing to disclose CM experiences, influenced by discussions with peers who may have shared their own experiences. This peer support and the diminishing stigma surrounding CM may make adolescents more comfortable self-reporting these traumatic events (36). While numerous factors influence adolescents’ reporting of CM, it is equally important to consider potential reasons from the perspective of adults. Adults, having undergone more life experiences, may perceive their CM as less significant or may not identify it as CM at all. This reinterpretation or underestimation of CM events by adults could contribute to the observed differences between the two age groups.

The high prevalence of certain PDs among both adolescents and adults, as indicated by the prominence of AVO, BOR, and OBC PDs in this age group, can be understood through several factors. Especially, our clinicians had been aware of the elevated prevalence of BOR PD (37) and other PDs in adolescent clinical populations. Adolescence is a period marked by identity exploration and instability. BOR PD traits, including unstable self-identity, impulsive behaviors, and intense interpersonal relationships (38), may align with the normative developmental processes of this age group (39). Adolescents often experience intense and rapidly shifting emotions. BOR PD, characterized by emotional instability, may manifest more prominently during this phase due to the emotional turbulence inherent to adolescence (40). Recognizing the potential emergence of BOR PD traits in adolescents is essential. Early intervention can help adolescents develop healthier coping strategies and emotional regulation skills. Offering evidence-based treatments, such as dialectical behavior therapy or mentalization-based treatment (41), can be effective in addressing BOR PD traits in adolescents (42).

The observed differences in CM and PD profiles between adolescents and adults as predictors of psychotic and non-psychotic disorders highlight the complex interplay of various factors. In adolescents, the prominence of SCHT and ANT PD traits in predicting psychotic disorders may reflect the emergence of these traits during adolescence. Such traits might be indicative of early signs of psychosis (8). The association of EN with psychotic disorders might be linked to the emotional intensity experienced during adolescence. EN, combined with the vulnerability inherent in psychotic disorders, could exacerbate the risk of developing such conditions (43). BOR PD traits, along with experiences of PN, predict non-psychotic disorders. Adolescents may be more susceptible to borderline traits. PN may contribute to emotional dysregulation and an increased risk of non-psotic disorders. In adults, SCHT PD traits and SA predict psychotic disorders. The persistence of SCHT traits into adulthood, combined with SA, could indicate a more enduring vulnerability to psychotic disorders (44).

The finding that probands with a family history of mental illness reported higher levels of CM, including physical abuse, sexual abuse, and emotional neglect, compared to those without such a history, is notable. This suggests a potential intergenerational transmission of adversity within families affected by mental illness. The increased prevalence of CM among individuals with a family history may reflect a complex interplay of genetic predisposition, environmental factors, and familial dynamics. These findings underscore the importance of considering family history as a significant factor in the assessment and treatment of psychiatric disorders, as it may provide valuable insights into the etiology and trajectory of illness. Furthermore, addressing the impact of childhood adversity in individuals with a family history of mental illness may be crucial for developing targeted interventions aimed at breaking the cycle of intergenerational transmission and improving long-term outcomes.

While this study offers valuable insights into our research topic, it is essential to acknowledge certain limitations that influence the interpretation and application of the findings. First and foremost, the adoption of a cross-sectional design necessitates caution when attempting to establish causal relationships between CM, PD, and clinical diagnoses. This approach provides a snapshot of data at a specific point in time, making it challenging to determine the directionality of observed associations. Additionally, the study may be susceptible to recall bias, as participants were required to recollect past events and experiences. Recall bias can arise due to memory inaccuracies or selective memory, leading to errors or incomplete information that may impact the validity and generalizability of the findings. Lastly, it is important to note that the findings may lack generalizability due to the utilization of a single-center sample. The results may not fully represent broader populations, and caution should be exercised when extrapolating these findings to different settings or demographics.

## Conclusion

This study reveals critical age-related differences in PDs and CMs, shedding light on their implications for psychiatric diagnoses. These findings underscore the importance of tailoring clinical interventions to address the unique needs of adolescents. Future research in this area should delve deeper into the underlying mechanisms that drive these age-related differences in PDs and CMs. Additionally, longitudinal studies can help establish causal relationships and provide a more comprehensive understanding of how these factors influence the development and course of psychiatric disorders over time.

## Data availability statement

The raw data supporting the conclusions of this article will be made available by the authors, without undue reservation.

## Ethics statement

The studies involving humans were approved by Shanghai Mental Health Cener Research Ethics Committee. The studies were conducted in accordance with the local legislation and institutional requirements. Written informed consent for participation in this study was provided by the participants’ legal guardians/next of kin.

## Author contributions

TZ: Writing – review & editing, Writing – original draft, Validation, Resources, Methodology, Funding acquisition, Conceptualization. WW: Writing – original draft, Investigation, Data curation. YC: Data curation, Methodology, Formal analysis, Investigation, Writing – original draft, Writing – review & editing. QH: Writing – original draft, Investigation, Data curation. YW: Writing – review & editing, Formal analysis, Data curation. LX: Writing – review & editing, Investigation. XT: Writing – review & editing, Methodology, Formal analysis. YH: Writing – review & editing, Data curation. HL: Writing – review & editing, Formal analysis. ZW: Writing – review & editing, Investigation, Data curation. TC: Writing – review & editing, Formal analysis. RW: Writing – review & editing, Data curation. CA: Writing – review & editing, Visualization, Validation, Supervision, Resources, Funding acquisition. JW: Writing – review & editing, Writing – original draft, Visualization, Validation, Supervision, Resources, Methodology, Funding acquisition, Conceptualization.
